# Shenmai injection suppresses multidrug resistance in MCF-7/ADR cells through the MAPK/NF-κB signalling pathway

**DOI:** 10.1080/13880209.2020.1742167

**Published:** 2020-04-06

**Authors:** Lin Yang, Chengda Zhang, Jiaoting Chen, Sheng Zhang, Guixuan Pan, Yanfei Xin, Lin Lin, Zhenqiang You

**Affiliations:** a Zhejiang Academy of Medical Sciences, Hangzhou Medical College, Hangzhou, China; b Collaborative Innovation Center of Yangtze River Delta Region Green Pharmaceuticals, Zhejiang University of Technology, Hangzhou, China

**Keywords:** P-glycoprotein, apoptosis, rhodamine 123

## Abstract

**Context:**

Shenmai Injection (SMI) is usually used to treat atherosclerotic coronary heart disease and viral myocarditis in China. However, the effect of SMI on multidrug resistance has not been reported.

**Objective:**

To investigate the reversal effect of SMI in adriamycin (ADR) resistant breast cancer cell line (MCF-7/ADR) and explore the related molecular mechanisms.

**Materials and methods:**

The effect of SMI (0.25, 0.5, 1 mg/mL) to reverse chemoresistance in MCF-7/ADR cells was elucidated by MTT, HPLC-FLD, DAPI staining, flow cytometric analysis, western blotting. At the same time, *in vivo* test was conducted to probe into the effect of SMI on reversing ADR resistance, and verapamil (10 μM) was used as a positive control.

**Results:**

The results showed that the toxicity of ADR to MCF-7/ADR cells was strengthened significantly after treated with SMI (0.25, 0.5, 1 mg/mL), the IC_50_ of ADR was decreased 54.4-fold. The intracellular concentrations of ADR were increased 2.2-fold (*p* < 0.05) and ADR accumulation was enhanced in the nuclei (*p* < 0.05). SMI could strongly enhance the ADR-induced apoptosis and increase intracellular rhodamine 123 accumulation in MCF-7/ADR cells. Additionally, a combination of ADR and SMI (5 mg/kg) could dramatically reduce the weight and volume of tumour (*p* < 0.05). Furthermore, the results revealed that SMI might reverse MDR via inhibiting ADR-induced activation of the mitogen-activated protein kinase/nuclear factor (NF)-κB pathway to down-regulated the expression of P-glycoprotein (P-gp).

**Discussion and conclusions:**

SMI could potentially be used to treat ADR-resistance. This suggests possibilities for future clinical research.

## Introduction

Considered as a vital treatment for cancer, chemotherapy drugs are usually effective in the early stage. However, in the middle and late stages, the efficacy of chemotherapy drugs is significantly reduced, and some types of cancer are resistant to multidrug chemotherapy. This phenomenon is called multidrug resistance (MDR). MDR is regarded as a major barrier to the efficacy of chemotherapy treatment (Rivera 2010). Many studies have suggested that the overexpression of P-glycoprotein (P-gp), a binding target of antitumour drugs which is located on the cell membrane, is the most frequent cause of MDR (Gottesman and Pastan 1993). P-gp can efflux intracellular drugs via ATP hydrolysis for energy, which results in the failure of chemotherapy (Binkhathlan and Lavasanifar 2013). Therefore, identifying effective MDR reversal agents to maintain the efficacy of chemotherapeutic agents is urgently needed.

Shenmai injection (SMI), derived from Sheng Mai San, is a prescription consisting of the root of *Panax ginseng* C.A. Meyer (Araliaceae) and *Ophiopogon japonicus* (Linn. f.) Ker-Gawl. (Asparagaceae). It was approved by the China Food and Drug Administration for the treatment of heart failure in 1995. SMI is widely used for the treatment of atherosclerotic coronary heart disease and chronic pulmonary heart disease in China (Li et al. 2011). SMI has high antioxidant activity and anticancer effects, balances blood pressure, and eliminates surplus free radicals (Yu et al. 2014; Shi et al. 2015; Zhou et al. 2017). Nevertheless, there were few reports that ginsenosides could reverse MDR except 20(*S*)-Rh_2_ (Zhang et al. 2012a, 2012b). Rh_2_ was a kind of ginsenosides in *P. ginseng* but lost during the preparation of SMI (Haijiang et al. 2003). Ginsenosides have similar chemical structures, and therefore may have similar pharmacological properties. Hence, we hypothesized that there are other effective reversal MDR components in SMI by detecting its efficacy of reversing MDR. Breast cancer is the most commonly diagnosed cancer among women worldwide (Siegel et al. 2017). In the present study, we systemically evaluated whether SMI could reverse the MDR of the breast cancer MCF-7/ADR cell line and investigated the mechanism by which it produces reversal effects.

## Materials and methods

### Reagents

SMI (10 mL per bottle) was provided by Chiatai Qingchunbao Pharmaceutical Co., Ltd. (Hangzhou, China). One bottle contains 2 g of crude drugs (1 g of *Panax ginseng* and 1 g of *Ophiopogon japonicus*). Adriamycin was purchased from Wuhan far co Creation Technology Co., Ltd. (Wuhan China). Verapamil was purchased from Sigma-Aldrich Co. (St. Louis, MO, USA). All ginsenosides (purity > 98%) were purchased from Source leaf Biotechnology Co., Ltd. (Shanghai, China).

High-performance liquid chromatography (HPLC)-grade methanol was purchased from Sigma-Aldrich (St. Louis, MO, USA). Deionized water was prepared via the Milli-Q system (Millipore Corporation, Billerica, MA, USA) and was used throughout the experiment. All other reagents and solvents were commercially available and of analytical grade.

### UHPLC analysis of ginsenosides in SMI

**Figure 1.**
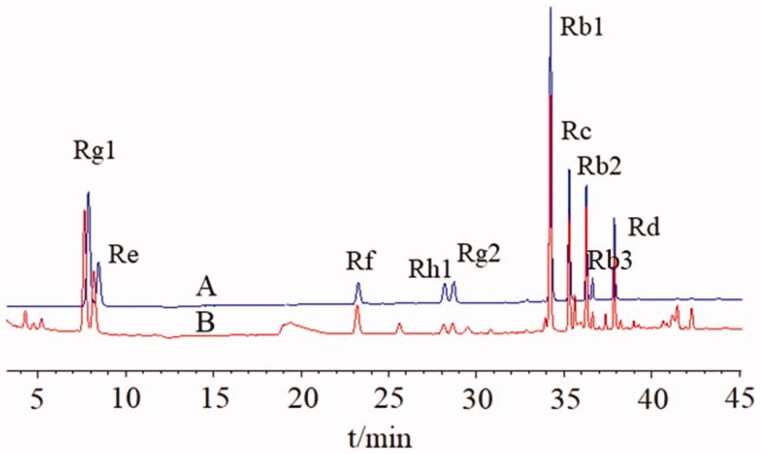
(A) UHPLC chromatograms of eleven standards and (B) the Shenmai injection sample.

Ultra High Performance Liquid Chromatography (UHPLC) was employed to achieve simultaneous quantitation of 10 main ginsenosides (Rg1, Re, Rf, Rh1, Rg2, Rb1, Rc, Rb2, Rb3, Rd) in SMI. The analysis was performed on a C18 (2.1 mm × 50 mm, 1.8 μm) column. The column temperature was 30 °C. The mobile phase consisted of acetonitrile (A) and water (B) at a gradient of 0.3 mL/min (0–10 min, 19% A; 10–32 min, 19–28% A; 32–39 min, 28–36% A). The UV detection wavelength was set at 203 nm, and injection volume was 1 μL. Retention time was shown in Figure 1. The contents of ginsenosides in the SMI were: Rg1, 0.150 mg/mL; Re, 0.093 mg/mL; Rf, 0.036 mg/mL; Rh1, 0.011 mg/mL; Rg2, 0.013 mg/mL; Rb1, 0.310 mg/mL; Rc, 0.099 mg/mL; Rb2, 0.081 mg/mL; Rb3, 0.010 mg/mL; Rd, 0.042 mg/mL, respectively.

### Cell culture

Human breast cancer cell line MCF-7 and Adriamycin-resistant cells derived from MCF-7 (MCF-7/ADR) were obtained from the Zhejiang University of Technology (Hangzhou, China). All cell lines were cultured in RPMI 1640 supplemented with 10% foetal bovine serum, 100 unit/mL penicillin, and 100 μg/mL streptomycin at 37 °C under 5% CO_2_. To maintain the MDR phenotype, the medium of MCF-7/ADR cells was supplemented with 1 μg/mL ADR. All cells were cultured in drug-free medium for more than 2 weeks until used in *in vitro* assay.

### Cytotoxicity and MDR reversal assays

3-(4,5-dimethyl-2-thiazolyl)-2,5-Diphenyl-2H-tetrazolium bromide (MTT) colorimetric assay was performed to evaluate cell viability and examine the effects of SMI on the sensitivity of MCF-7/ADR cells to adriamycin. Cells were plated onto 96-well plates (5 × 103 cells/well) overnight and incubated with various concentrations of adriamycin and SMI (equivalent to *P. ginseng* 0.25, 0.5, 1 mg/mL) for 24 h. Subsequently, 20 μL of MTT solution (5 mg/mL) was added to each well and incubated at 37 °C for 4 h. After carefully discarding the medium, the intracellular MTT stain was dissolved in 150 μL dimethyl sulfoxide, then the absorbance of samples was measured at 570 nm with enzyme-linked immunosorbent assay reader (BioTek Instruments, Inc, Winooski, USA). The degree of resistance was presented as the IC_50_ drug concentration. The reversal fold was calculated by dividing the IC_50_ of ADR alone by that of ADR plus SMI in MCF-7/ADR cells. All analyses were performed in triplicate.

### Apoptosis evaluation using flow cytometry

For apoptosis analysis, MCF-7/ADR cells were seeded onto 6-well plates (5 × 105 cells/well) overnight and were incubated with 5 μM ADR in the absence or presence of SMI (0.25, 0. 5, 1 mg/mL) for 24 h. Cells were harvested and washed twice with ice-cold PBS. Apoptosis analysis was evaluated with the Annexin V/FITC Apoptosis Detection Kit (Nanjing Jiancheng Bio-engineering Institute, Nanjing, China) following the manufacturer’s instructions. The cells resuspended with Binding Buffer. Afterwards, 5 μL of Annexin V-FITC and 10 μL of PI were added to the cell suspension. After gently mixing, the mixture was incubated for 5 min at room temperature in the dark. The cell samples were analyzed with BD FACSCalibur™ Flow Cytometer (BD Bioscience, San Jose, CA).

### Intracellular rhodamine 123 accumulation

The accumulation of intracellular Rhodamine 123 was used to evaluate the function of P-gp as a transporter. MCF-7 and MCF-7/ADR cells were seeded onto 12-well plates and cultured overnight. Subsequently, the cells were pre-treated with or without SMI (0.25, 0.5, 1 mg/mL) at 37 °C for 4 h and then incubated with 5 μM Rhodamine 123 for 2 h under 5% CO_2_ in the dark at 37 °C. Verapamil (10 μM) was used as positive control. Then, cells were collected, centrifuged, washed twice with ice-cold PBS, and resuspended in 0.4 mL PBS. The accumulation of intracellular Rhodamine 123 was detected using BD FACSCalibur™ Flow Cytometer (BD Bioscience, San Jose, CA). All analyses were performed in triplicate.

### Cellular retention assay

MCF-7 and MCF-7/ADR cells were seeded onto 12-well plates. After ∼90% confluence, the cultured cells were pre-treated with SMI (0.25, 0.5, 1 mg/mL) for 4 h, followed by the addition of 5 μM adriamycin. Verapamil (10 μM) was used as positive control. After incubation for another 2 h, stopped the retention. Then, the cells were digested and washed twice with ice-cold PBS. Cells were lysed via three freeze–thaw cycles, and protein concentrations were measured via the Bradford method. Adriamycin was determined by HPLC-FLD. All experiments were conducted in triplicate.

### Distribution of ADR in MCF‐7/ADR cells

The MCF-7/ADR cells were seeded onto 6-well plates (5 × 105 cells/well) and cultured overnight. Subsequently, the cells were pre-treated with gradient concentrations of SMI (0.25, 0.5, 1 mg/mL) for 4 h and incubated with 5 μM ADR for 30 min. Verapamil (10 μM) and 20(*S*)-Rh2 (10 μM) were used as positive control. Afterwards, discarded the cell culture medium, and the cells were washed twice with ice-cold PBS. Then, 4% paraformaldehyde was used to fix the cells for 15 min. After the paraformaldehyde was removed, the cells were washed twice and stained with 4′,6-diamidino-2-phenylindole for nuclei staining. The Olympus fluorescence microscope (Tokyo, Japan) was used to detect ADR intracellular distribution, and the blue and red fluorescence was monitored with different excitation lights for the nuclei and ADR, respectively. The fluorescence intensity of ADR in nuclei quantifications was analyzed via the Image J software.

### Western blot analysis

Aiming at clarifying the possible mechanism of SMI in suppressing multidrug resistance in MCF-7/ADR cells, western blot analysis was performed. The cells were seeded onto 6-well plates (5 × 105 cells/well) overnight. Subsequently, the cells were incubated with 5 μM of ADR in the absence or presence of SMI (0.25, 0.5, 1 mg/mL) for 24 h, and 10 μM of Rh2 was used as positive control. Cells were lysed with RIPA Lysis Buffer (Byotime Biotechnology, China). The samples of tumour issue were then washed with PBS and homogenated in RIPA buffer on ice (1 g/mL). Total protein concentration was measured via the BCA Protein Assay Kit. Protein samples were separated by 12% sodium dodecyl sulphate polyacrylamide gel electrophoresis (SDS-PAGE) and transferred onto a PVDF membrane. Then, 5% skim milk was used to block PVDF membranes at room temperature for 2 h. Afterwards, the membranes were probed with primary antibodies anti-P-gp, anti-JNK, anti-ERK, anti-p38, a nti-p65 (diluted 1:1000, CST, Beverly, MA, USA), anti-IκBα or anti-tubulin (diluted 1:1000, Santa Cruz Biotechnology, DBA Italy) at 4 °C overnight. After being washed with Tris-buffered saline/Tween-20 (TBS/T), the membranes were incubated with secondary antibody horseradish peroxidase conjugated goat anti-mouse IgG or goat anti-rabbit IgG (diluted 1:2000, Bioker Biological Technology, China) for 2 h at room temperature. Immunoreactive proteins were visualized with enhanced chemiluminescence. The density of the immunoreactive band quantifications were analyzed via Image J software.

### MCF7/ADR xenografts mice model

All mouse protocols were approved by the Animal Care and Use Committee of Zhejiang Academy of Medical Sciences (Hangzhou, Zhejiang, China). Male BALB/C nude mice (4–6 weeks old, 16–18 g) were obtained from Shanghai Slack Laboratory Animal Co., Ltd., the qualification certificate number is 1710100007. The 1 × 107 MCF‐7/ADR cells were suspended in 100 μL of serum free RPMI 1640 medium (mixed with Matrigel), then injected into the right flank of nude mice. Tumour growth was observed every 3 days until the diameter of subcutaneous tumours reached 0.5 cm. After screening out the mice of oversize or too small tumour, the rest were randomly assigned to four treatment groups (*n* = 4 per group): control (0.1 mL normal saline, iv, once every 3 day), SMI (5 mL/kg, once every 3 day), ADR (5 mg/kg, once every 3 day), SMI + ADR (5 mL/kg SMI + 5 mg/kg ADR, once every 3 day). And for the last group, SMI was given 1 h before ADR was injected. The tumour volume was monitored with callipers and calculated as V = 1/2 × length × width^2^ (mm^3^). After the treatments, the mice were sacrificed and the tumour bulks were harvested for further studies.

### Statistical analysis

Data was expressed as the mean ± SEM. Differences among groups fitted the normal distribution and homogeneity of variances were analyzed by One-way ANOVA. Histograms and scatter diagrams were produced by using PRISM 6.0 (GraphPad Software, USA). *p*<0.05 was considered to be statistically significant.

## Results

### SMI reversed adriamycin resistance in MCF-7/ADR cells

**Figure 2.**
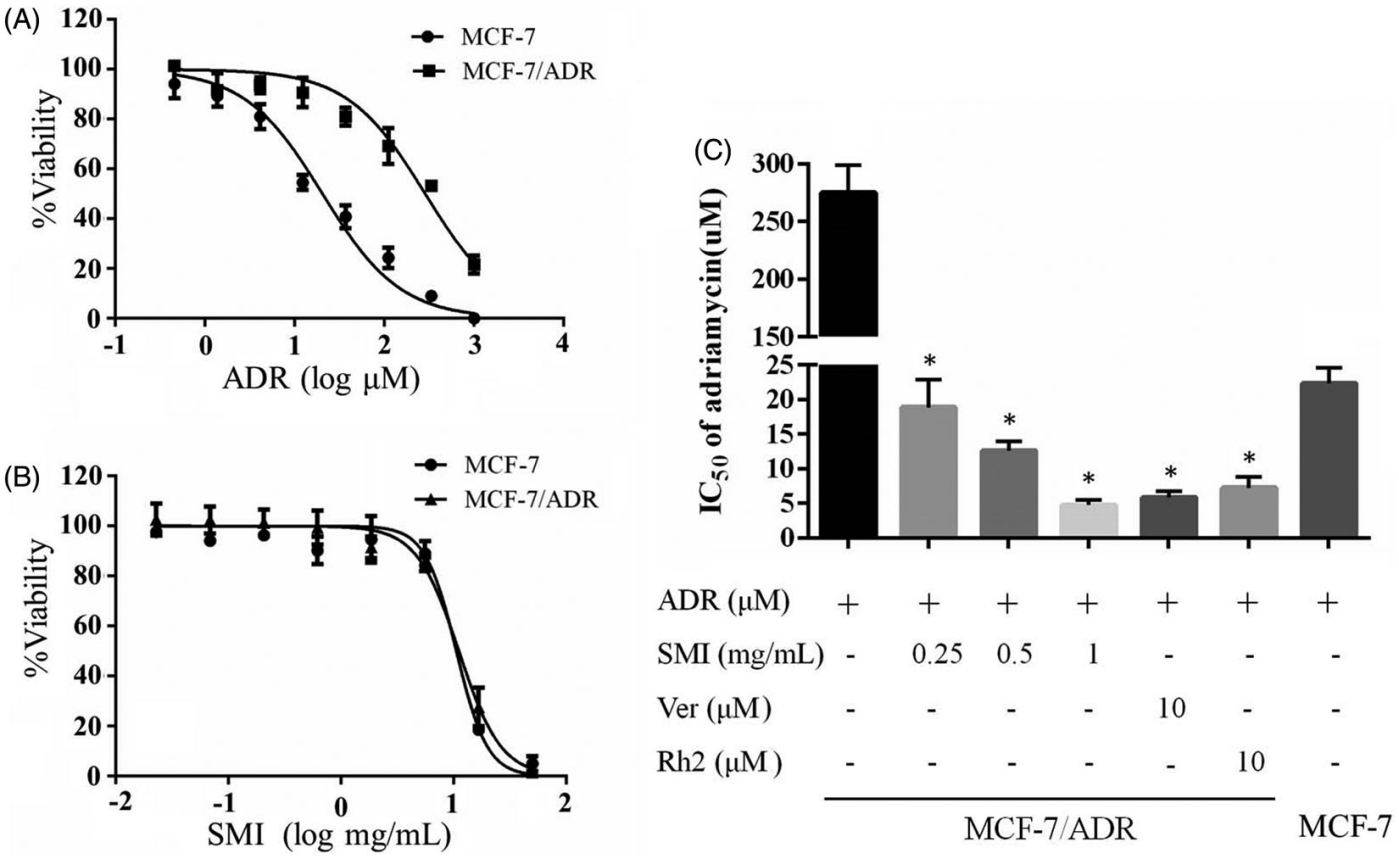
Effects of SMI on the viability of breast cancer cells. (A,B) Cytotoxicity of ADR (A) SMI (B) in MCF-7 and MCF‐7/ADR cells. (C) Effects of SMI on the sensitivity of MCF-7/ADR cells towards ADR were examined by MTT. Data are the mean ± SEM of three independent experiments. *p < 0.05 versus control.

The results of MTT assay indicated that the ADR IC_50_ values of MCF-7 cells and MCF-7/ADR cells were 20.46 and 286.28 μM, which suggests that MCF-7/ADR cells were more resistant to ADR than MCF-7 cells for approximately 14-fold (Figure 2(A)). Figure 2(B) showed that more than 95% of MCF-7/ADR cells were viable when treated with 1.8 mg/mL SMI. These results suggested that SMI potently reversed the resistance to ADR in MCF-7/ADR cells under non-toxic conditions. The IC_50_ values of ADR were decreased significantly when MCF-7/ADR cells were co-incubated with SMI (0.25, 0.5, 1 mg/mL) for 24 h (Figure 2(C)). In addition, 1 mg of SMI took almost the same effect as 10 μM of P-gp inhibitor verapamil. By contrast, the IC_50_ values of ADR remained unchanged when MCF-7 cells were co-incubated with various concentrations of SMI.

### SMI promoted ADR-induced apoptosis in MCF-7/ADR cells

**Figure 3.**
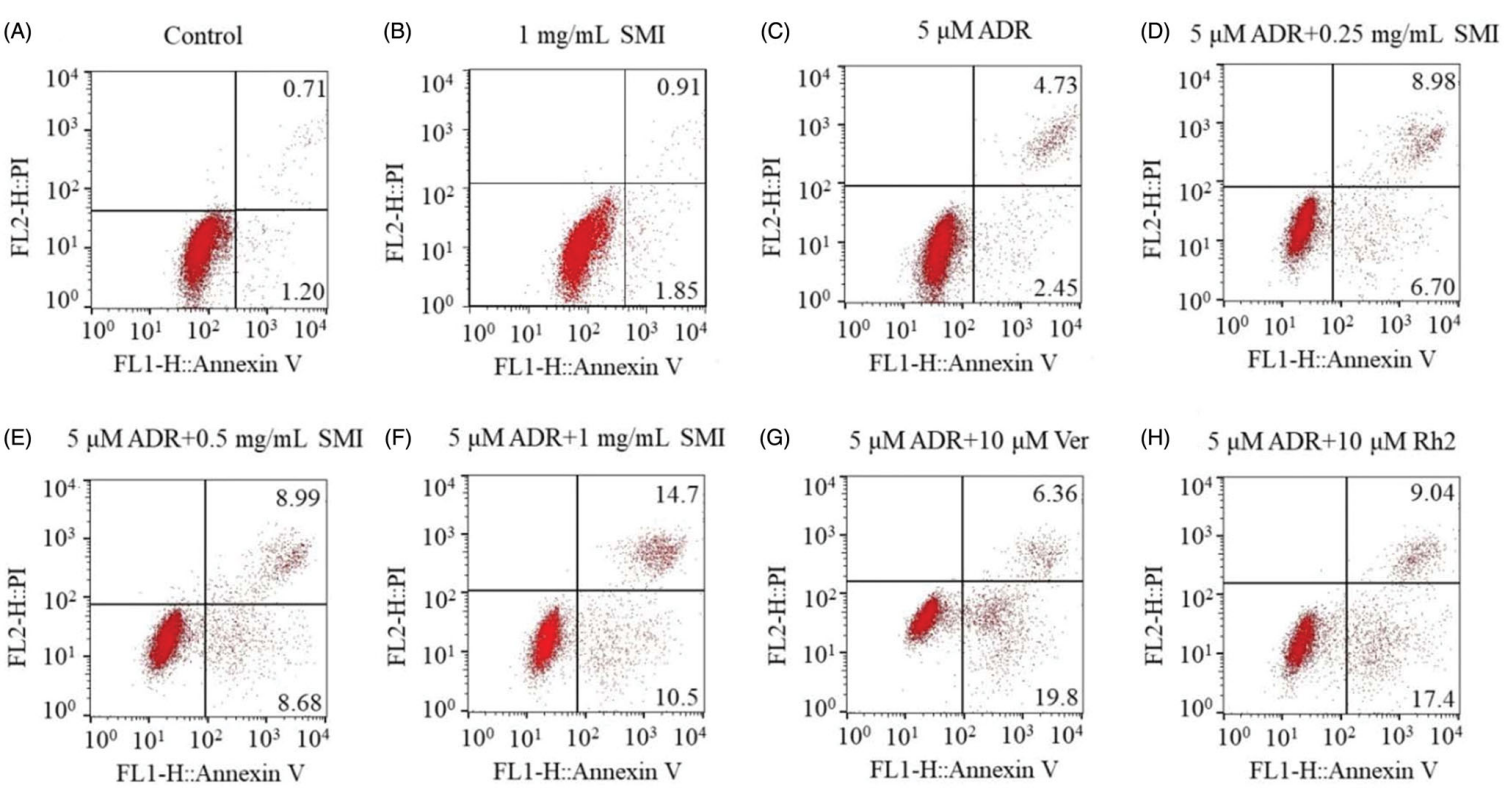
Flow cytometric analysis of ADR induced apoptosis of MCF-7/ADR cells. Early apoptotic cells are in the lower right quadrant, late apoptotic cells are in the upper right quadrant.

To investigate the effects of SMI on ADR-induced apoptosis, a flow cytometer was adopted in our study. As shown in Figure 3, treatments with various concentrations of SMI resulted in an obvious increase in the rate of ADR-induced apoptosis in MCF-7/ADR cells compared with that in the ADR treatment alone group. In addition, verapamil (10 μM) and 20(*S*)-Rh2 (10 μM), a new potential MDR reversal agent in *P. ginseng*, have similar effects with those in the SMI (1 mg/mL) treatment group.

### SMI increased intracellular accumulation and uptake rate of ADR in MCF-7/ADR cells

**Figure 4.**
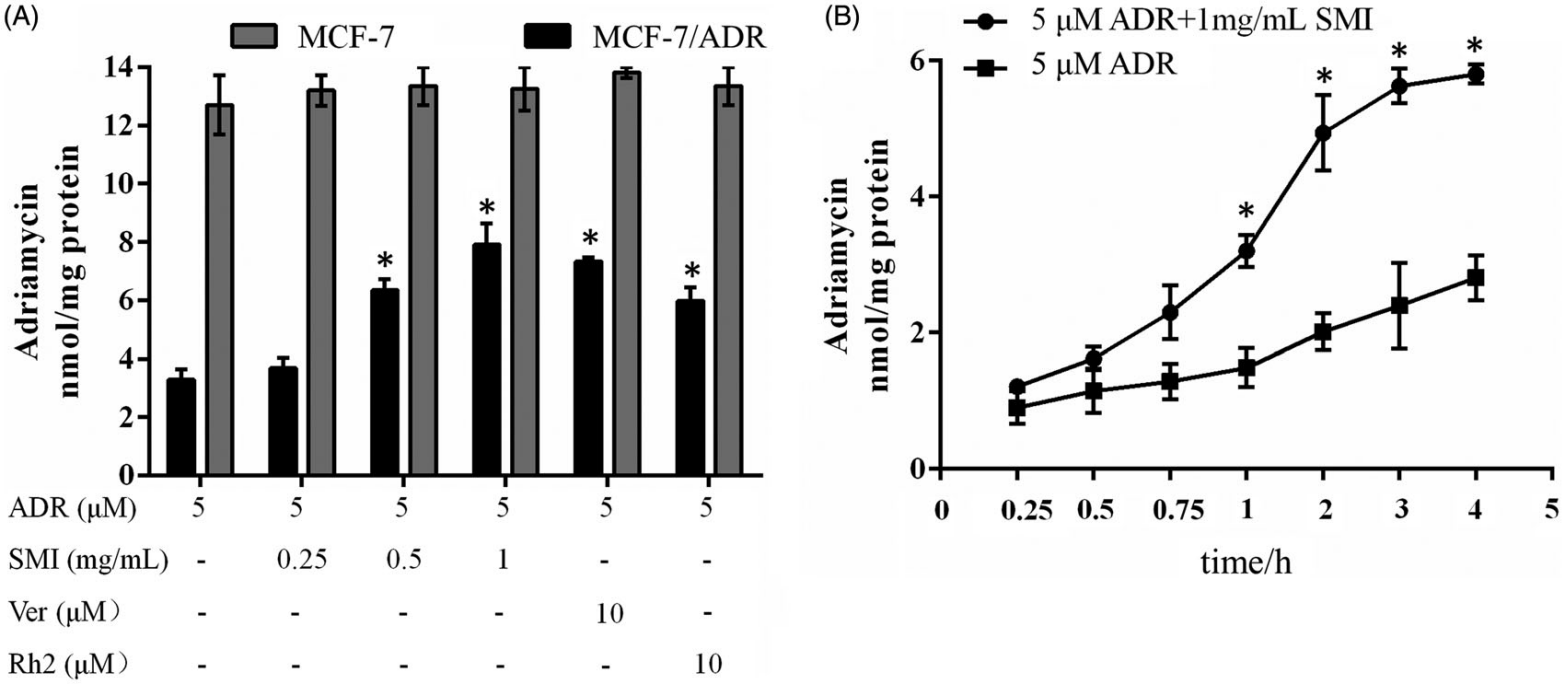
Effects of SMI on the accumulation and uptake rate of ADR in MCF-7 and MCF-7/ADR cells. (A) Effects of SMI on accumulation of ADR in MCF-7 and MCF-7/ADR cells. (B) Effects of SMI on uptake rate of ADR in MCF-7/ADR cells. Data are the mean ± SEM of three independent experiments. *p < 0.05, compared with ADR group.

The accumulation of ADR in MCF-7/ADR cells was 3.86-fold lower than that in MCF-7 cells, which is the main cause of drug resistance in MCF-7/ADR cells (Figure 4(A)). After treating with SMI, the accumulation of ADR in MCF-7/ADR cells increased significantly (*p* < 0.05). However, the cellular accumulation of ADR in MCF-7 cells was hardly increased in the absence or presence of SMI (*p* > 0.05). We further compared the MDR reversal effects of SMI with 10 μM of verapamil and 10 μM of 20(*S*)-Rh2. The potency of SMI (1 mg/mL) was almost the same as that of the positive controls verapamil and Rh2.

The ADR uptake in MCF-7/ADR cells was also altered by SMI (Figure 4(B)). After pre-treatment with SMI for 1 h, the intracellular ADR concentrations increased more rapidly in MCF-7/ADR cells compared with the ADR alone treatment group and reached a maximum within 4 h. These results indicate that SMI can improve intracellular accumulation and uptake rate of ADR in MCF-7/ADR cells.

### SMI increased distribution of ADR in MCF‐7/ADR cells

**Figure 5.**
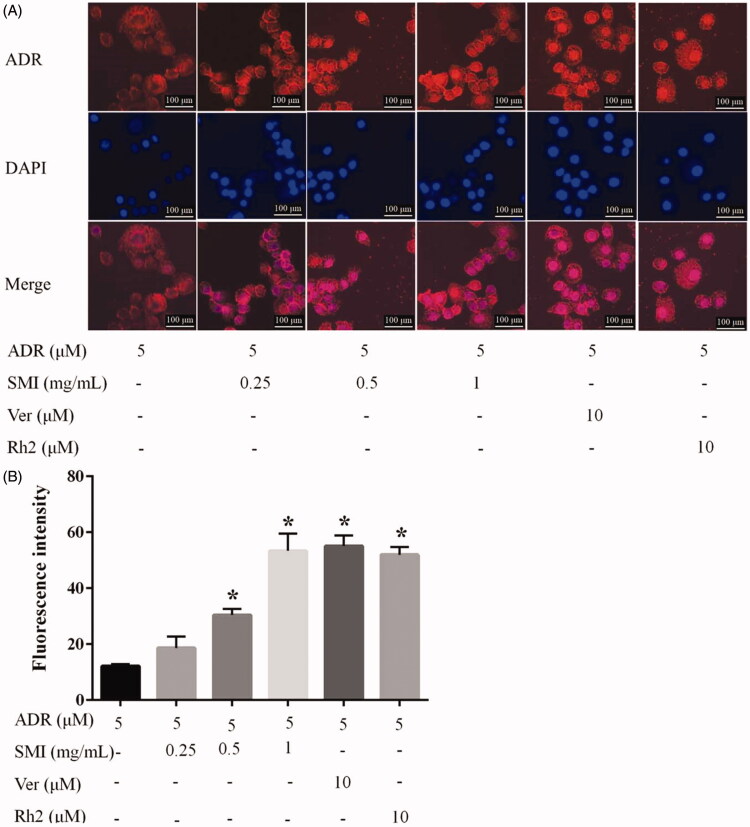
Effects of SMI on ADR distribution in MCF‐7/ADR cells. (A) Images of ADR distribution in MCF7/ADR with various concentrations of SMI (0.25, 0.5, 1 mg/mL) pretreat for 4 h and then incubated with 5 μM ADR for 30 min. Cell nuclei were stained with DAPI. Scale bar, 100 μm. (B) Fluorescence quantification of the ADR accumulation in nuclei. Data are the mean ± SEM of three independent experiments. *p < 0.05, compared with ADR control group.

As a DNA-damaging agent, ADR intercalates into nuclear DNA. The effects of SMI on the nuclear accumulation of ADR were evaluated qualitatively and quantitatively in MCF-7/ADR cells. As shown in Figure 5(A), few ADR (red fluorescence) appeared in the MCF-7/ADR cells after 5 μM ADR treatment for 45 min in the absence of SMI, which primarily gathered around the nucleus. Several ADR were located in the nuclei, and the drug accumulation in these cells was increased significantly after pre-treatment with SMI. The results of fluorescence quantification in nuclei are shown in Figure 5(B). The fluorescence intensity was increased significantly by SMI (0.5, 1 mg/mL), compared with ADR group (*p* < 0.05), and 1 mg/mL SMI has the same effect as verapamil and 20(*S*)-Rh2.

### SMI inhibited P-gp activity and expression in MCF-7/ADR cells

**Figure 6.**
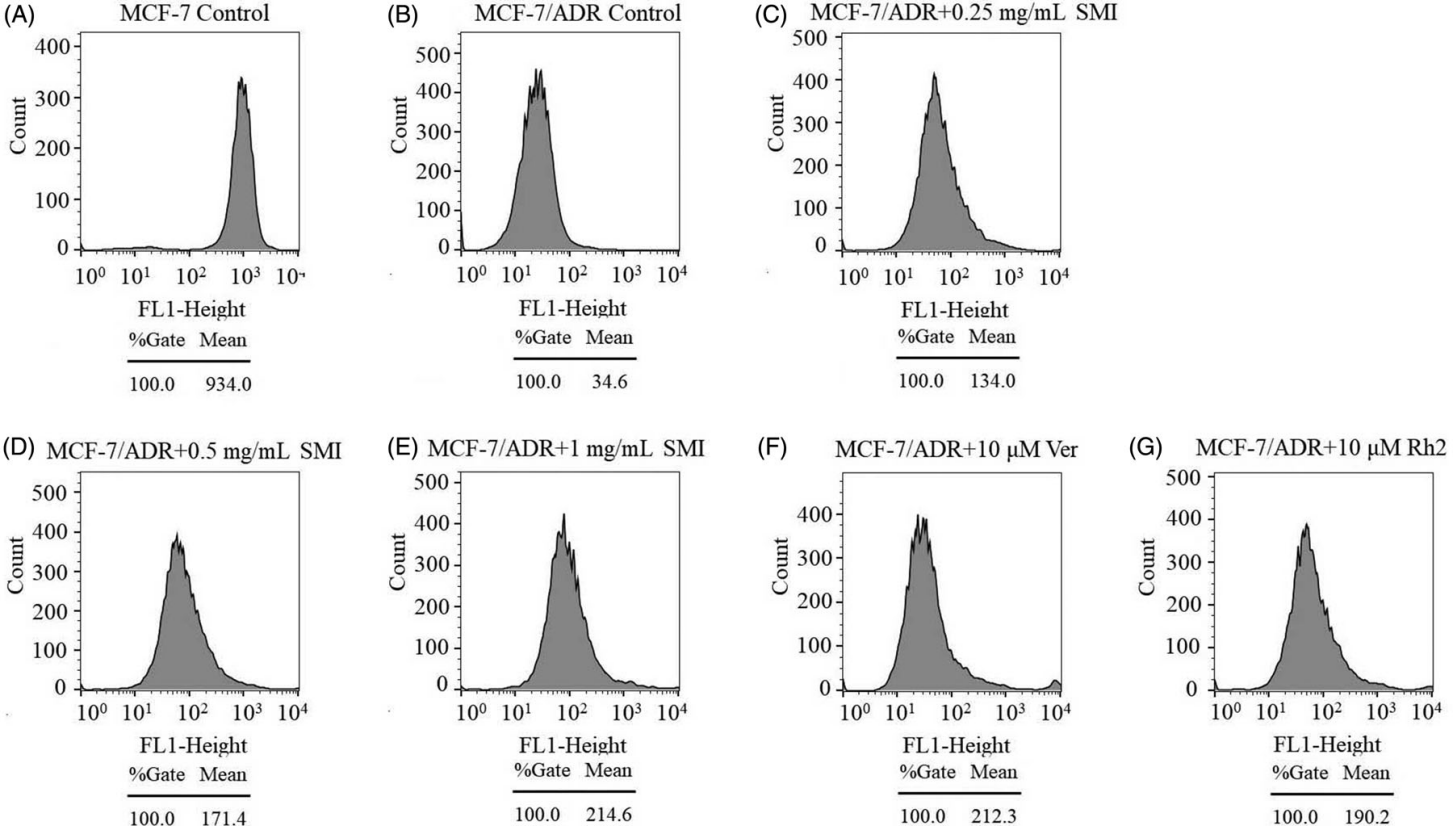
Effects of SMI on the intracellular accumulation of Rho-123 in MCF-7/ADR cells.

To assess the efflux function of P-gp, the effects of SMI on intracellular Rho-123 accumulation were evaluated using flow cytometry (Zhou et al. 2016). The results of the mean fluorescence intensity of intracellular Rho-123 are shown in Figure 6. The level of Rho-123 accumulation in MCF-7 cells was much higher than that in MCF-7/ADR cells, which also indicated that P-gp is overexpressed in MDR cell lines. The mean intracellular fluorescence intensity of Rho-123 was significantly enhanced with SMI pre-treatment in MCF-7/ADR cells. In addition, SMI (1 mg/mL) has a comparable potency to the P-gp inhibitor verapamil (10 μM). Therefore, it could be inferred that SMI might directly interact with the binding sites of P-gp to inhibit its efflux activity.

**Figure 7.**
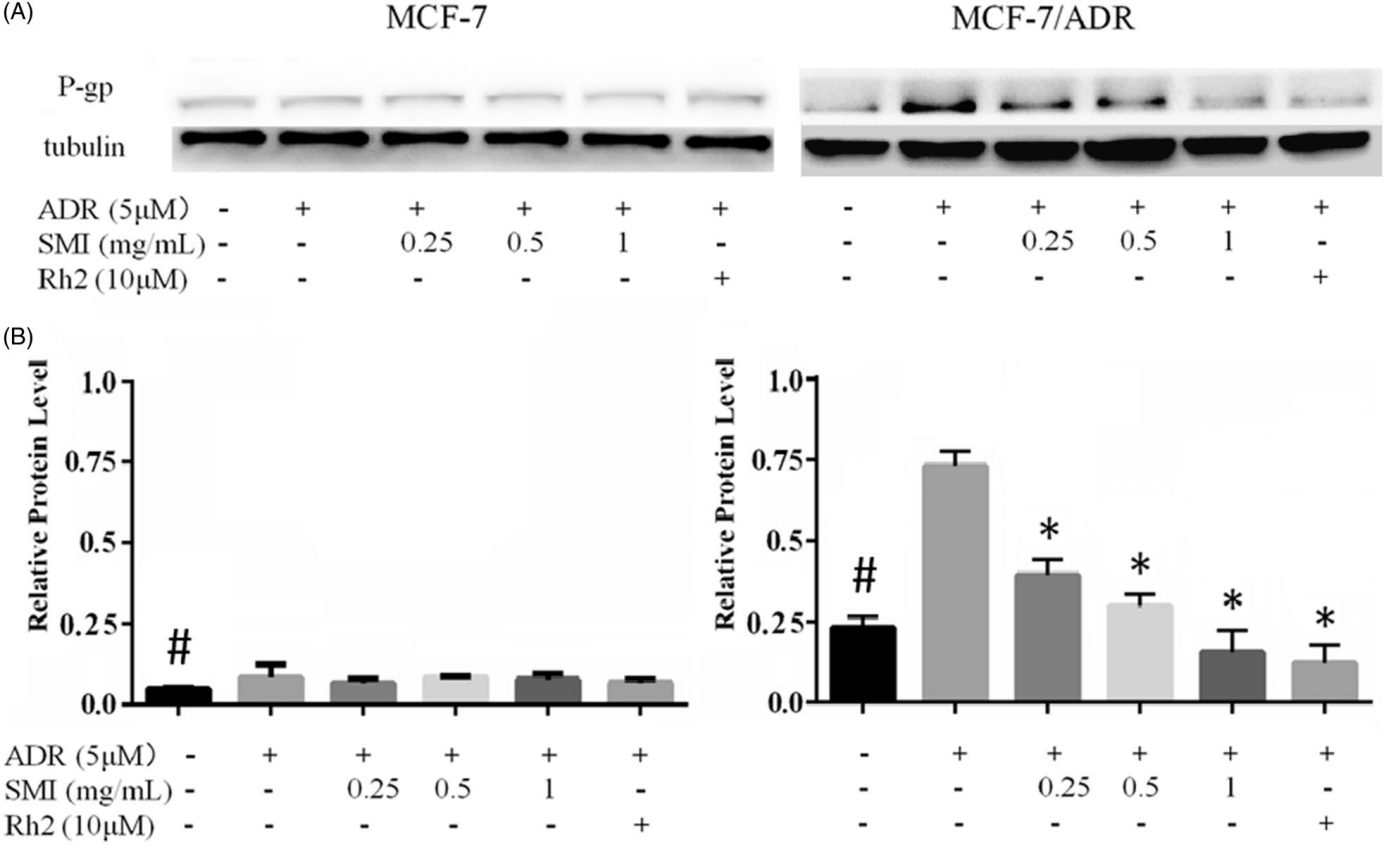
Effects of SMI on protein expression of P‐gp. (A) Western blot results of P-gp expression in MCF-7 and MCF-7/ADR. (B) The bar graph that shows the protein level of P-gp was normalized to the level of tubulin, the loading control. Data are the mean ± SEM of three independent experiments. #*p* < 0.05 versus MCF-7 control group; *p < 0.05 versus ADR group.

However, in addition to inhibiting its function, SMI was doubted to reverse P-gp-mediated MDR by reducing its expression. Therefore, we further investigated the effects of SMI on the expression of P-gp. As a result, western blot analysis indicated that the expression of P-gp was significantly weaken in MCF-7 cells compared with that in MCF-7/ADR cells (Figure 7). Simultaneously, the expression of P-gp in MCF-7/ADR cells was significantly increased in the ADR alone group compared with that in the blank control group, while the expression of P-gp was apparently decreased after co-treatment with SMI in MCF-7/ADR cells. However, no difference was observed between the control group and the SMI treatment groups in MCF-7 cells. Furthermore, the potency of SMI (1 mg/mL) inhibition of ADR-induced P-gp expression in MCF-7/ADR cells was comparable with that in 20(*S*)-Rh2 (10 μM).

### SMI reversed MDR in MCF-7/ADR cells via inhibiting the MAPK/NF-κB pathway

**Figure 8.**
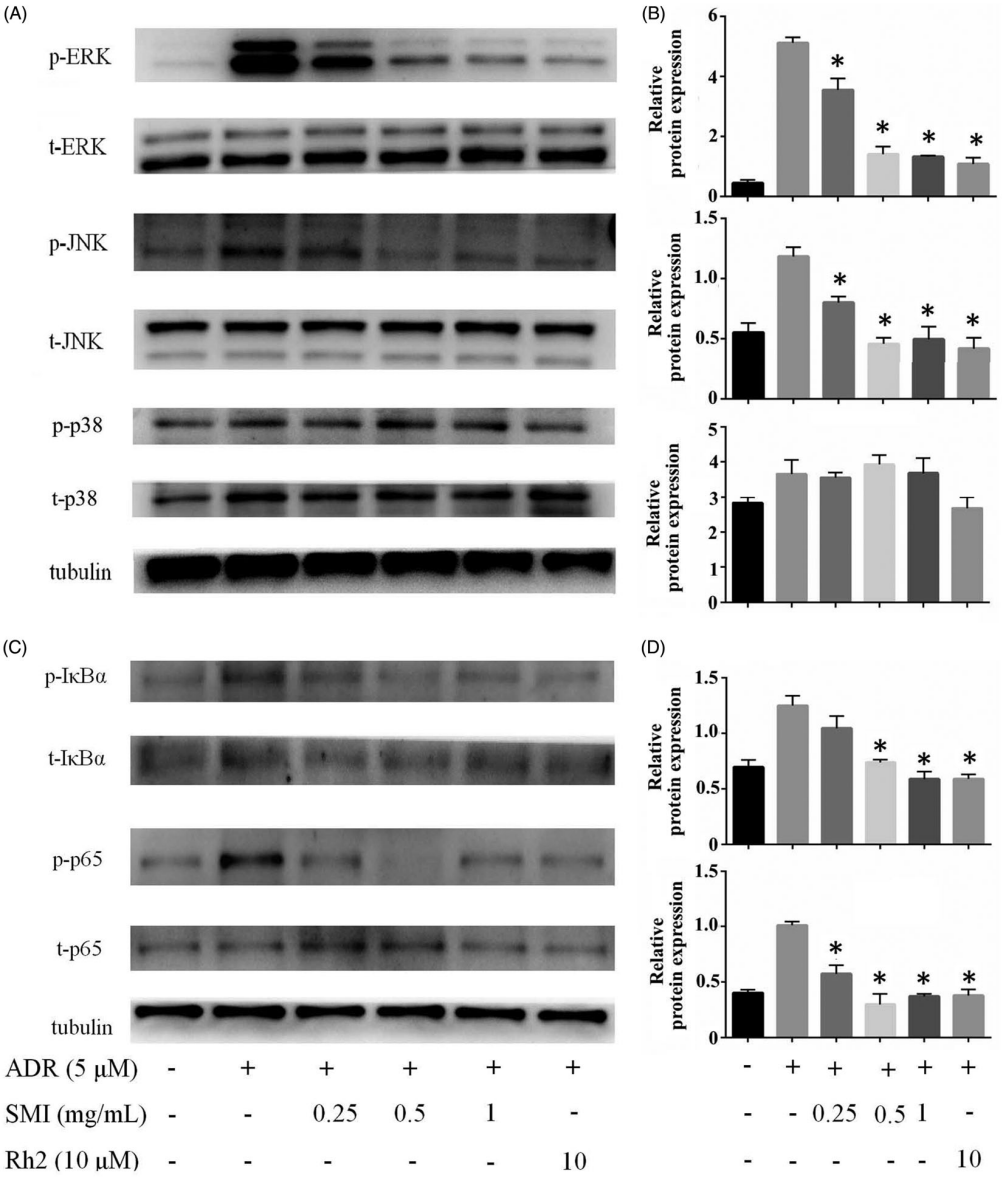
SMI reversed MDR of MCF-7/ADR cell via inhibition of MAPK/NF-κB pathway. (A,B) SMI inhibited the activation of MAPK pathway in MCF-7/ADR cells. (C,D) SMI inhibited the activation of NF-κB pathway in MCF-7/ADR cells. The protein level of p-ERK, p-JNK, P-IκBα, p-p65 was normalized to the level of tubulin, the loading control. Data are the mean ± SEM of three independent experiments. *p < 0.05 versus ADR group.

We further explored the molecular mechanism for the down-regulation of P-gp by SMI. As shown in Figure 8, the phosphorylation of ERK and JNK was activated by 5 μM ADR treatment for 24 h in MCF-7/ADR cells compared with the control group. Nevertheless, after MCF-7/ADR cells were co-treated with SMI, the phosphorylation of ERK1/2, and JNK was remarkably inhibited. However, in the present study, SMI had no significant effect on the phosphorylation of p-p38. And no significant effect was observed on the expression of total ERK and JNK after SMI treatment. The phosphorylation of p65 and IκBα was increased by ADR exposure but was remarkably suppressed by SMI treatment. We found that the expression levels of total p65 and IκBα were not altered by treatment of ADR or SMI. Similar effects of 20(*S*)-Rh2 were revealed in the positive control group. The results indicated that SMI moderates the ADR-induced expression of P-gp via the inhibition of the MAPK/NF-κB pathway.

### SMI reversed MDR in vivo

**Figure 9.**
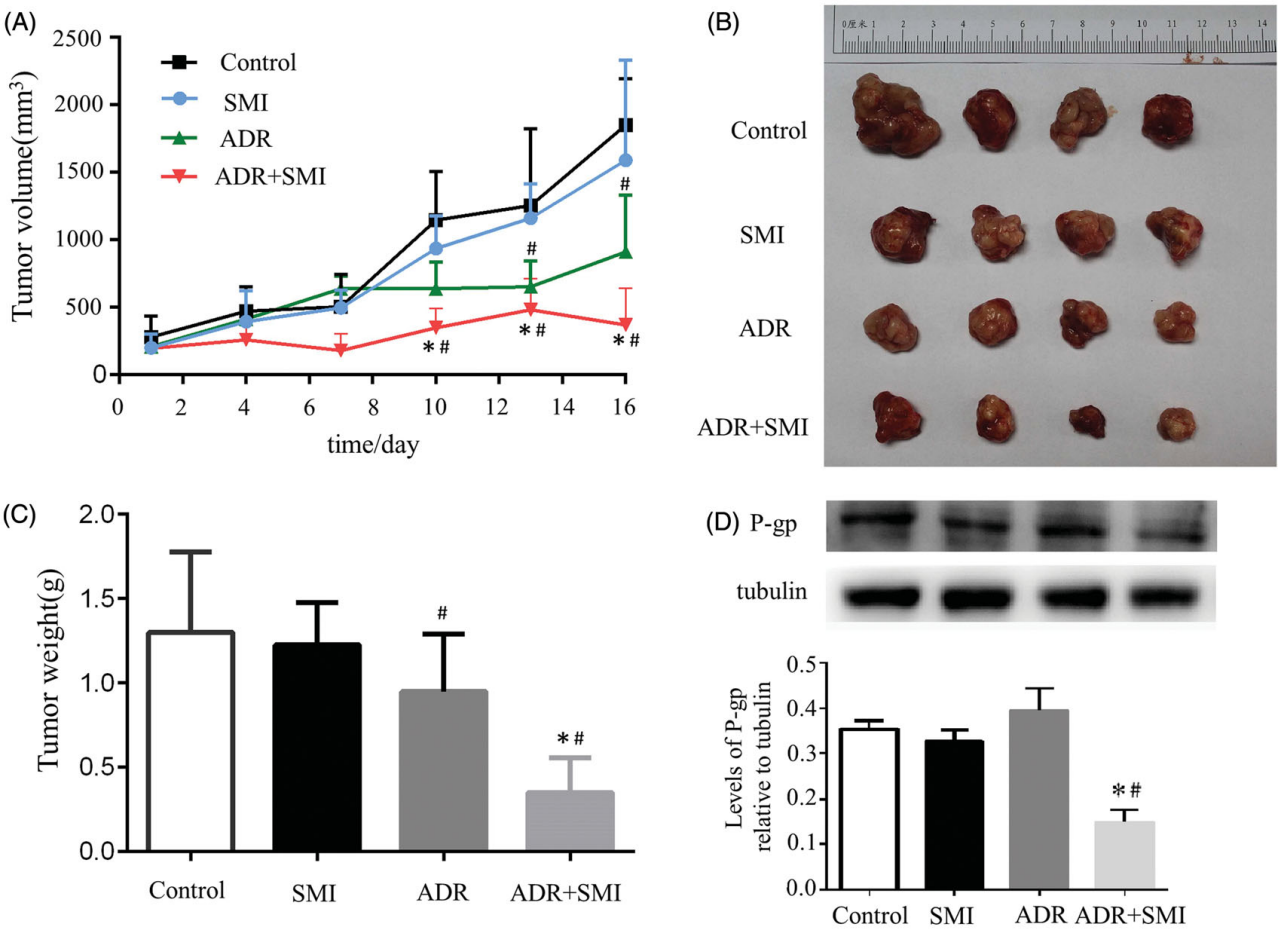
*In vivo* combined effect of SMI and ADR on MCF-7/ADR xenografts. (A) Tumour volume during drug treatments. (B,C) The overview and weight of the isolated MCF-7/ADR tumours in the end of the treatments. (D,E) Western blot results of P-gp expression in MCF-7/ADR xenografts. Data are the mean ± SEM of three independent experiments. #*p* < 0.05 versus control group; *p < 0.05 versus ADR group.

To evaluate whether Shenmai injection has the effect of reversing adriamycin resistance *in vivo*, the MCF-7/ADR cell xenograft in nude mice was established. The results showed that both ADR alone and SMI combined with ADR inhibited tumour growth with smaller tumour volumes and lower tumour weights at the end of the treatment, compared with control group (*p* < 0.05) (Figure 9(A,B)). However, the combination of SMI and ADR had the most significant inhibitory effect (*p* < 0.05, compared with ADR group) (Figure 9(C,D)). Moreover, western blotting results also indicated that SMI combined with ADR markedly reduced P-gp expression in the tumour tissue but that ADR did not.

## Discussion

Chemotherapy is an important treatment for breast cancer. However, its effectiveness is affected by MDR. Although many compounds, such as verapamil and progesterone (Shapiro et al. 2001), were reported to be efficient in reversing MDR, whose side effects limit their own clinical application. Hence, developing effective reversal agents with minimal toxicological effects to conquer MDR is extremely urgent (Nobili et al. 2006).

SMI has been used for the clinical treatment of heart failure in China. The efficacies of SMI are based on various ginsenosides (Yu et al. 2017), which are primarily extracted from *P. ginseng*. These compounds consist of Rg1, Re, Rf, 20(*S*)-Rh_1_, 20(*S*)-Rg_2_, Rb_1_, Rc, Rb_2_, Rb_3_, and Rd. The total content of ginsenosides in SMI was 0.845 mg/mL. SMI has antioxidant effects because of its components (Lu et al. 2009). Few reports indicated that ginsenosides, except 20(*S*)-Rh_2_, could reverse MDR (Zhang et al. 2012a, 2012b). Rh_2_ is a ginsenoside in *P. ginseng* that is lost during the preparation of SMI (Haijiang et al. 2003). In this study, we explored whether there exist other effective reversal MDR components in SMI by detecting its efficacy in reversing MDR. In our experiments, the efficacy of SMI as a potent reversal agent for overcoming the MDR of MCF‐7/ADR cells was evaluated. The potency of 10 μM 20(*S*)-Rh_2_, which is approximately twice higher than the concentration of total ginsenosides in SMI (1 mg/mL), was compared with the potency of SMI. As a result, SMI was found to effectively reverse MDR, and its potency was stronger than that of 20(*S*)-Rh_2_ at the same concentration.

The results of MTT assay demonstrated that MCF-7/ADR cells were 14-fold less sensitive to ADR than MCF-7 cells. A significant reduction in the IC_50_ of ADR was found in MCF-7/ADR cells when treated with SMI, but no significant reversal effect in the parent MCF-7 cells was observed (Figure 2). These results were confirmed by the apoptosis assay. The early apoptosis of MCF-7/ADR cells were sharply increased after treatment of SMI compared with that of the ADR alone groups (Figure 3), which indicates that SMI showed an excellent potential to reverse MDR.

As a DNA-damaging agent, the nucleus is the target of ADR (Finn et al. 2011). Previous studies reported that P-gp is overexpressed not only on the cell membrane but also on the nuclear membrane (Munteanu et al. 2006; Barot et al. 2013), which may prevent ADR entry into the nucleus to produce its cytotoxic effect and provoke MDR (Li Y et al. 2018). To investigate the effects of SMI on the accumulation of ADR in the nucleus, the nuclear accumulation of ADR was evaluated qualitatively and quantitatively. The results demonstrated that ADR accumulation was enhanced in the nuclei of SIM treated MCF7/ADR cells. Similar results were observed with barbigerone, which reverses MDR (Li X et al. 2018).

P-gp overexpression is the main cause of MDR, which weakens the cytotoxicity by pumping intracellular drugs out (Iakusheva et al. 2014). In general, reversal agents increased the efficacy of chemotherapies by directly inhibiting the function of P-gp or regulating its expression (Hennessy and Spiers 2007). The mitochondrial fluorescent dye Rho-123 is a high-affinity substrate for P-gp, which can be used to evaluate the transport function of P-gp (Jouan et al. 2014). Previous studies showed that 20(*S*)-Rh_2_ is a non-competitive inhibitor of P-gp (Zhang et al. 2010). Therefore, the reversal effect of SMI on MDR with its P-gp inhibitory effect was correlated in this study. Our results showed that the level of Rho-123 accumulation in MCF-7/ADR cells was augmented after SMI treatment compared with the blank control groups. Concomitantly, the assay results of cellular retention of ADR showed that SMI could enhance ADR accumulation and uptake in MCF-7/ADR cells. The potency of SMI (1 mg/mL) was similar to that of the positive control verapamil (10 μM) and Rh_2_ (10 μM). These results indicate that SMI can obviously inhibit the function of P-gp as a transporter.

Previous reports revealed that the inhibition of the MAPK/NF-κB pathway is a possible mechanism for the reversal of MDR in MCF-7/ADR cells. Several studies indicated that activation of the NF-κB signalling pathway could promote the expression of MDR1 gene, contributing to the expression of P-gp (Krishnamurthy et al. 2012; Fang et al. 2014). In the present study, western blot analysis results showed that compared with the blank control group, ADR can significantly activate the MAPK signalling pathway by increasing the phosphorylation of ERK, and JNK and then P65 to activate the downstream NF-κB signalling pathway. However, compared with the ADR alone group, SMI significantly inhibited the phosphorylation of ERK1/2, and JNK.

Numerous studies have demonstrated that the modulation of autophagy is one of the most promising therapeutic strategies to confer MDR. MAPKs, mainly including ERK1/2 and JNK, play a major role in regulating various forms of autophagy (Sridharan et al. 2011). A previous report indicated that increased phospho-JNK levels induced autophagy and promoted cell death in resistant cells (Mukherjee et al. 2017). In the present study, SMI significantly inhibited MAPKs. For example, the phosphorylation of ERK1/2 and JNK altered the sensitivity to ADR in MCF-7/ADR cells. The findings suggested that the regulation of autophagy by MAPKs may partly contribute to the effects of SMI in reversing MDR of MCF-7/ADR cells. However, this suggestions requires further investigation.

## Conclusions

The present study first confirmed that SMI enhanced the sensitivity of breast cancer cells to chemotherapy by inhibiting P-gp function and expression. SMI is a promising MDR-reversal agent with low toxicity. The reversal effect of SMI in tumour xenograft models indicated that combination of SMI with ADR may be important in surmounting clinical resistance in cancer chemotherapy. However, further studies are needed to confirm our findings.
